# Ketoconazole for the Treatment of Docetaxel-Naïve Metastatic Castration-Resistant Prostate Cancer (mCRPC): A Systematic Review

**DOI:** 10.31557/APJCP.2021.22.10.3101

**Published:** 2021-10

**Authors:** Raden Indra Tresnanda, Sawkar Vijay Pramod, Ferry Safriadi

**Affiliations:** *Department of Urology, Hasan Sadikin Academic Medical Center, Universitas Padjajaran Bandung, Indonesia. *

**Keywords:** Ketoconazole, Corticosteroid, mCRPC, PSA, PFS, Response

## Abstract

**Objective:**

This systematic review aimed to determine the efficacy of ketoconazole in the treatment of metastatic castration-resistant prostate cancer (mCRPC).

**Materials and Methods:**

A literature search was performed on four databases of PubMed, Google Scholar, Cochrane Database of Systematic Reviews, and Directory of Open Access Journals (DOAJ). The initial search resulted in 602 articles, which were progressively eliminated based on duplication, irrelevancy, and unsuitable methodology. A total of seventeen articles were included in the final analysis, including four randomized controlled trials, nine retrospective cohorts, and four prospective cohorts, with a total population of 1,095 patients. A 200-400 mg, tid dose of ketoconazole was used in these studies along with corticoid replacement therapy with hydrocortisone, 20-30 mg in the morning and 10-20 mg in the evening, or prednisone, 5 mg, bid.

**Results:**

Based on our findings, 8 out of 17 studies reported PSA decrease of >50% in approximately half of the population, with a more significant PSA response at 400 mg ketoconazole dosage, and the average progression-free survival (PFS) of 2.6-14.5 months, or time to progression of 3.2-6.7 months.

**Conclusion:**

Ketoconazole with corticosteroid could be an effective alternative for the treatment of mCRPC with a satisfactory PSA response and disease progression.

## Introduction

Prostate cancer is the most common malignancy in men. Worldwide, prostate cancer is the third most common cancer after lung and breast cancers, and the second most common cancer in men after lung cancer (Bray et al., 2018; Rawla et al., 2019). This cancer has a significant morbidity and mortality burden, with annual losses of 7.1 million disability-adjusted life-years (DALYs) and more than 358.000 deaths in 2018 (Rawla et al., 2019; Global Burden of Disease Cancer Collaboration; 2019). Metastatic castration-resistant prostate cancer (mCRPC) is a subtype of prostate cancer having a poor prognosis. The average survival rate of this subtype is less than two years after diagnosis contributing to a large number of deaths from prostate cancer (Frieling et al., 2015). CRPC is defined by prostate cancer progression and signified by an increase of tumor volume during radiographic evaluation and continuous increase in prostate-specific antigen (PSA) levels on three different occasions, despite a castrate level of testosterone (less than 50 ng/dL) (Fong et al., 2012).

The standard treatment for CRPC is androgen deprivation therapy with the addition of secondary hormonal manipulation, chemotherapy, and radiotherapy. Androgen deprivation is usually achieved through the use of anti-androgenic agents, including abiraterone or enzalutamide, in addition to androgen synthesis inhibitors, such as ketoconazole and corticosteroid replacement therapy. The standard chemotherapeutic agents for CRPC include alkaloids, such as docetaxel, or anthracenediones, such as mitoxantrone (Teo et al., 2019). However, chemotherapy is well known for the side effects, such as diarrhea, nausea and vomiting, risk of infection, and bleeding (American Cancer Society medical and editorial content team, 2019).

In Indonesia, malignancies are one of the costliest diseases in the health care system. A study by Budiarto and Sugiharto (2013) stated that cancer took the second-highest cost in the Indonesian Case-Based Groups (INA-CBGs) after cardiovascular diseases with a total cost per patient of 4,805 USD (American Cancer Society medical and editorial content team, 2019; Budiarto et al., 2012). Moreover, not all prostate cancer chemotherapy agents are covered by national health insurance or private insurance, limiting therapeutic options for these patients. Although there are no available official epidemiologic data of prostate cancer in Indonesia currently, Hasan Sadikin Academic Medical Center reported that 39.3% of patients with prostate cancer treated in the hospital experienced metastatic progression. A report by the Indonesian Ministry of Health showed that approximately 10% of prostate cancer cases in Indonesia progressed to CRPC. Several new therapeutic agents for CRPC, such as sipuleucel-T, enzalutamide, radium-233, abiraterone, estramustine, and cabazitaxel, are currently not approved for use in Indonesia, thus they are not covered by the Indonesian national health insurance system. Therefore, a safe, efficient, and economical alternative is needed for mCRPC patients in Indonesia (Komite Penanggulangan Kanker Nasional., 2016).

**Figure 1:**
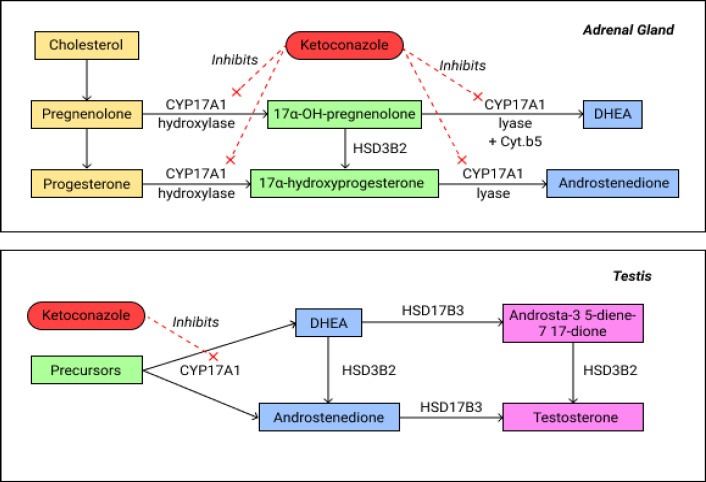
The Role of Ketoconazole in Inhibiting Gonadal Steroid Hormone Synthesis (Patel., 2018)

Ketoconazole is a synthetic imidazole antifungal agent commonly used in the treatment of dermatophytosis or systemic fungal infections, including blastomycosis, histoplasmosis, paracoccydiomycosis, coccydiomycosis, and chromomycosis. Ketoconazole is a nonspecific cytochrome P450 inhibitor, which may inhibit gonadal and adrenal steroid synthesis (Sinawe et al., 2020). Furthermore, ketoconazole inhibits CYP17A1, a catalyst for two key reactions in the production of gonadal and extragonadal steroid hormones (androgen and testosterone), as shown in Figure 1 (Sinawe et al., 2020). Considering the limitations of chemotherapy in Indonesia, including the availability of drugs and facilities, the lack of oncological expertise, the high cost of chemotherapy, and the higher toxicity, our study was performed to determine the effectiveness of ketoconazole for the treatment of mCRPC. 

## Materials and Methods

This systematic review was done through searching PubMed, Cochrane Database of Systematic Reviews, Google Scholar, and Directory of Open Access Journals (DOAJ). The literature search was carried out with no time limitations using the keywords of ketoconazole, metastatic castrate-resistant prostate cancer, and mCRPC. A literature search was also performed on synonyms of mCRPC, including advanced prostate cancer, androgen-independent prostate cancer, hormone refractory prostate cancer, and rogressive androgen-independent prostate cancer. Studies that used ketoconazole after docetaxel chemotherapy were excluded from the analysis. This systematic review was performed according to the PRISMA guidelines (Moher et al., 2010). 

**Figure 2:**
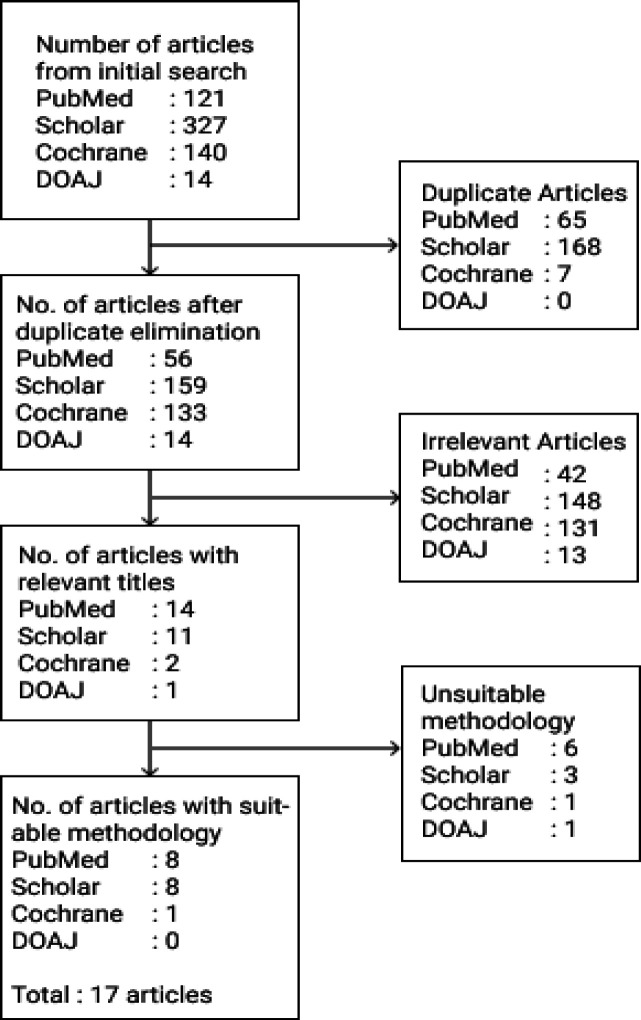
Article Elimination Flowchart

All articles obtained from the initial search were analyzed in four steps. The first step was filtering the articles based on the searched keywords. The second step was analyzing the title and abstract based on the inclusion and exclusion criteria. In the third step, the generalized text was analysed based on the inclusion and exclusion criteria. In the fourth step, cross-referenced articles in other systematic reviews or studies. The number of eliminated articles is described in Figure 2.

## Results

Out of seventeen included studies, four studies were randomized controlled trials (RCT), nine studies were retrospective cohorts, and four studies were prospective cohorts. All studies were done on a population of patients with prostate cancer resistant to metastatic castration and naïve to docetaxel. All of the studies used ketoconazole, 200 to 400 mg, tid as the primary intervention. Fifteen of the seventeen studies used hydrocortisone, prednisone, or prednisolone as an alternative therapy to prevent corticosteroid deficiency due to long-term use of ketoconazole.

*PSA response and disease progression*

All of the analyzed studies reported a significant decrease in serum PSA (>50%) in mCRPC patients receiving ketoconazole. Eight studies reported PSA response in >50% of patients. Scholz et al., (2005) reported a PSA response >75% in 44% of patients. While Chiang et al., (2012) reported a greater decrease in PSA in the group receiving 400 mg ketoconazole, compared to the group receiving 200 mg ketoconazole. Based on disease progression, which was assessed by the duration of progression-free survival (PFS), Small et al., (1997) reported a median PFS of 4 months, Chiang et al., (2012) reported a PFS of 7.5-11.5 months, Keizman et al., (2012) a PFS of 8 months, Keizman et al., (2012) a PFS of 8 months, Lin (2012) a PFS of 2.6 months (0.5 to 8.6 months), Millikan et al., (2001) a PFS of 3,3 months, Taplin et al., (2009) a PFS of 14.5 months, and Yun et al., (2011) a PFS of 8 months (Barata et al., 2018; Chiang et al., 2012; Keizman et al., 2012; Lin et al., 2012; Millikan et al., 2001; Taplik et al., 2009; Yun et al., 2011; Scholz et al., 2005). 

**Figure 3:**
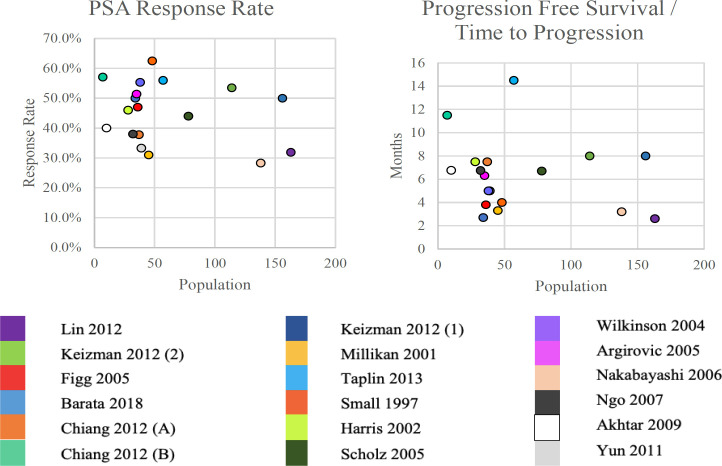
Scatterplot of PSA Response Rates and Progression-free Survival Durations from the Analyzed Studies

Several other studies reported various time to progression. For instance, Harris et al., (2002) reported time to progression of 30 weeks, Wilkinson and Chodak (2004) of 5 months, Argirovic et al., (2005) of 6.3 months, Nakabayashi et al., (2006) of 3.2 months, Akhtar et al., (2009) of 6.75 months, and Ngo et al., (2007) of 6.75 months. Figure 3 illustrates the results of these studies in detailes (Harris et al., 2002; Wilkinson et al., 2004; Argirović et al., 2005; Akhtar et al., 2009; Ngo et al., 2007; Nakabayashi et al., 2006).

*Ketoconazole Toxicity*

Ketoconazole generally shows a good safety profile, although its toxicity should be considered in managing mCRPC patients. Administration of systemic ketoconazole most commonly causes gastrointestinal side effects, including nausea, vomiting, constipation, abdominal pain, dry mouth, and discoloration of the tongue. Inhibition of steroid synthesis may result in adrenal insufficiency, which may consequently lead to orthostatic hypotension and side effects associated with sex hormones, including gynecomastia (Sinawe et al., 2020). Therefore, long-term administration of ketoconazole must be accompanied by replacement therapy using corticosteroids. The most severe symptoms of toxicity are generally associated with hepatotoxicity, which can cause jaundice, severe hepatitis, and liver failure (National Institute of Health, 2017).

Almost all studies used the National Cancer Institute Common Toxicity Criteria monitoring system to evaluate and analyze ketoconazole toxicity. The symptoms of grade 3-4 toxicity indicate poor tolerance by the patient and require cessation of the regimen as soon as possible. If the symptoms of toxicity have improved to degrees 1 or 2, the regimen can be restarted with a reduced dose. The summary of the analyzed studies is described in Table 1. 

**Table 1:** Summary of Analyzed Studies

Investigators, Year	Design	Subjects	Intervention	PSA Response and Disease Progression	Ketoconazole Toxicity
Small, et al. 1997, USA	Prospective cohort	48	Ketoconazole 3x400 mg + hydrocortisone 20mg-0-10mg	· > 50% PSA response in 62.5% patients · Median progression-free survival of 4 months · Median duration of response was 3.5 months (range 2-12 months) · Range time to reach PSA decrease was 3-10 months	· Hepatotoxicity in 2 patients · Nausea and anorexia in 3 patients · Congestive heart failure in 1 patient
Millikan, et al. 2001, USA	Randomized controlled trial	45	Ketoconazole 3x400 mg	· PSA response in 31% patients · Median time to progression was 3.3 months	· Dominated by gastrointestinal complaints · 1 patient had grade 4 hepatotoxicity
Harris, et al. 2002, USA	Prospective cohort	28	Ketoconazole 3x200mg + hydrocortisone 20mg-0-10mg	· 46% had a PSA decrease > 50% · Median duration of PSA response was 7.5 months	· Nausea in 29% patients · Dry skin in 18% patients · Liver effects in 8% patients
Wilkinson & Chodak 2004, USA	Retrospective cohort	38	Ketoconazole 3x300 mg + hydrocortisone 20mg-0-10mg	·> 50% PSA response in 55.3% patients · Median time to progression of 5 months (range 0-27 months) · Median duration of response was 6 months (range 3-48 months) · PSA response to ketoconazole can be identified within the first 6-8 weeks of therapy	· 31.6% patients reported toxicity symptoms
Argirovic 2005, Serbia	Retrospective cohort	35	Ketoconazole 3x400 mg + hydrocortisone 20mg-0-10mg	· PSA response to ketoconazole can be identified within the first 6-8 weeks of therapy · > 50% PSA response in 51.4% patients · Median time to progression of 6.3 months (range 0-27 months) · Mean duration of response was 6 months (range 3-48 months)	34.3% patients reported grade 1-2 toxicity symptoms
Figg, et al. 2005, USA	Randomized controlled trial	36	Ketoconazole 3x400 mg + hydrocortisone 30 mg-0-10 mg	· PSA response in 47% patients · Median progression free survival was 3.8 months · Median duration of response was 6.3 months	· 58% patients had grades 1 and 2 toxicity · 3% patients had grade 3 toxicity
Scholz, et al. 2005, USA	Retrospective cohort	78	Ketoconazole 3x200-400 mg + hydrocortisone 2x20 mg	· > 75% PSA response in 44% patients · Median time to progression was 6.7 months · Mean time to progression was 14.5 months	No severe toxicity reported
Nakabayashi, et al., 2006, USA	Prospective cohort	138	Ketoconazole 3x200-400 mg + hydrocortisone 40 mg/day OR prednisone 10 mg/day	· 50% PSA response in 28.3% patients · Median time to progression of 3.2 months (range 0.1-61 months) · Median duration of response in high dose ketoconazole was 3.6 months (range 0.5-32.2 months) · Median duration of response in low dose ketoconazole was 3 months (range 0.1-62.4 months)	· 10.9% patients experienced grade 3 toxicity · Fatigue in 13.5% patients · Anorexia in 6.0% patients · Nausea in 5.3% patients · Diarrhea in 4.5% patients
Ngo, et al., 2007, Singapore	Retrospective cohort	32	Ketoconazole 3x200 mg + hydrocortisone OR prednisolone (no reported dosage)	· 50% PSA response in 38% patients · Median duration of response was 6.75 months (range 2-14 months, mean 7.75 months) · Median time to reach PSA nadir was 3.5 months (range 1.5-11 months, mean 4.7 months)	· 56% reported toxicity symptoms · Abnormal liver function test in 41% patients
Akhtar 2009, Bangladesh	Retrospective cohort	10	Ketoconazole 3x200 mg	· >50% PSA response in 40% patients · Median duration of response of 6.75 months (range 2-14 months) · Median time to reach PSA nadir was 5.06 months (1.5-11 months)	50% patients reported grades 1 and 2 toxicity symptoms
Investigators, Year	Design	Subjects	Intervention	PSA Response and Disease Progression	Ketoconazole Toxicity
Yun, et al. 2011, South Korea	Retrospective cohort	39	Ketoconazole 3x200 mg + Prednisolone 2x5 mg	· PSA response in 33.3% patients · Median progression-free survival was 5 months · Early time to reach PSA decrease was within the 1st 4 weeks	· Nausea/vomitting in 20 (51.3%) patients · Anorexia in 15 (38.5%) patients · Facial edema in 11 (28.2%) patients · Gynecomastia in 3 (7.7%) patients · Liver toxicity in 9 (23.1%) patients
Chiang, et al., 2012, Taiwan	Retrospective cohort	44	Ketoconazole 3x200 mg + prednisolone (37) vs ketoconazole 3x400 mg + prednisolone (7)	· PSA response was 37.8% in 200 mg group and 57.1% in 400 mg group · Median time to progression was 7.5 months (range 1-87 months) in 200 mg group and 11.5 months (range 4-16.5 months) in 400 mg group · Median duration of response was 5.5 months (range 0.5-86 months) in 200 mg group and 9 months (range 2-13 months) in 400 mg group	· 9.1% patients had grade 1 or 2 toxicity · 2,3% patients had grade 3 elevated liver function
Keizman, et al. 2012, USA	Prospective cohort	114	Ketoconazole 3x200-400 mg + hydrocortisone 30 mg-0-10 mg	· 50% PSA response in 54% patients · 16 patients remained progression free with a median treatment time of 12 months · Median time to progression was 8 months (range 1-129 months, mean 12.8 months) · Median duration of response was 12 months (range 6-122 months, mean 29.9 months)	22% patients had grade 3 or 4 toxicity
Keizman, et al. (2) 2012, USA	Retrospective cohort	156	Ketoconazole 3x200-400 mg + hydrocortisone 30 mg-0-10 mg	· 50% PSA response in 50% patients · Median duration of response was 4 months (range 1-55 months, mean 6.8 months)	17% patients had grade 3 or 4 toxicity
Lin, et al. 2012, China	Retrospective cohort	163	Ketoconazole 3x200-400 mg + Prednisone 2x5 mg	· ≥ 50% PSA response in 31.9% patients · Median progression-free survival was 2.6 months (0.5–8.6 months) · Median time to reach PSA nadir was 1.4 months	· Abnormal liver function (9.2%) · Abnormal renal function (8.6%) · 1.8% had grade 3 toxicity
Taplin, et al., 2013, USA	Randomized controlled trial	57	Ketoconazole 3x400 mg + hydrocortisone 30 mg-0-10 mg	· Decrease in PSA in 56% patients · RECIST (response evaluation criteria in solid tumors) response in 30% patients · Median duration of response was 20 months · Median time to progression was 14.5 months	32% patients had grade 3 toxicity
Barata, et al. 2018	Randomized controlled trial	34	Ketoconazole 3x400 mg + hydrocortisone 30 mg-0-10 mg	· PSA response in 50% patients · Median time to failure was 2.7 months (0.2-32.8 months)	Most common adverse events: fatigue (76%), skin reactions (62%), lymphopenia (44%) and anemia (44%)

## Discussion

Secondary hormonal manipulation is a suitable therapeutic option to reduce the burden of disease and improve patients’ quality of life with CRPC. Ketoconazole is a nonspecific cytochrome P450 inhibitor, which inhibits CYP17A1, a catalyst for two key reactions in the production of gonadal and extragonadal steroid hormones, namelyandrogen and testosterone (Almassi et al., 2018; Moher et al., 2010). 

Another mechanism that may influence the pathogenesis of CRPC involves the effect of ketoconazole on T cell proliferation. In this case, cytochrome P450 regulates the influx of Ca^2^ + ions in T cells and causes inhibition of interleukin (IL) -2 synthesis and T24 cell proliferation (Jensen et al., 1999). 

The adrenal cortex produces 10-30% of serum androgens, including androstenedione, dehydroepiandrosterone (DHEA), and dehydroepiandrosterone sulfate (DHEAS). Free and serum testosterone suppression is obtained within 2 hours of oral ketoconazole consumption, with maximum suppression after 8 hours. The testosterone suppression effect of ketoconazole is rapid and reversible, and normal testosterone levels are restored within 24 hours. Inhibition of CYP17A1 in the androgen synthesis pathway plays a significant role in ketoconazole’s action against castration resistance. Androgen depletion therapy generally inhibits gonadal androgen synthesis, while ketoconazole inhibits both gonadal and adrenal androgen synthesis simultaneously (Patel et al., 2018). Furthermore, another source of androgen in prostate cancer is the de novo androgen synthesis in prostate cancer cells, mediated by CYPA1 and 3β-dehydroxysteroid dehydrogenase isoenzyme 1 (3βHSD1 or HSD3B1). HSD3B1 activity increases extragonadal androgen precursor conversion to intratumoral androgen, making ketoconazole’s extragonadal activity even more potent against intratumor androgen levels in patients with high HSD3B1 activity (Almassi et al., 2018; Moher et al., 2010).

Ketoconazole for the treatment of mCRPC can be administered with three approaches: (1) a low dose regimen of 200 mg, tid, increased to 400 mg, tid if PSA response is not achieved within 3 months; (2) a high-dose regimen of 400 mg, tid, lowered to 200 mg if the patient exhibited gastrointestinal intolerance or toxicity symptoms; or (3) an intermediate-dose of 300 mg, tid. The patients received ketoconazole on an empty stomach, either one hour before or two hours after eating, unless this resulted in nausea or gastrointestinal upset, in whom the ketoconazole was taken at mealtime (Harris et al., 2002; Milikan et al., 2001). Corticosteroids used for replacement therapy include hydrocortisone, 30 mg in the morning and 10 mg in the evening, or prednisone, 5 mg, bid (Millikan et al., 2001; Harris et al., 2002). In our study, in 17 extracted studies, it was concluded that ketoconazole significantly reduced more than fifty percent PSA value with or without corticosteroids or other adjuvants. It may be chosen for alternative ADT for metastatic CRPC patients especially in developing countries that cant afford abiraterone acetate or enzalutamide.

Inhibition of adrenal steroids by agents such as ketoconazole is the standard therapy for mCRPC, although the serological and radiographic benefits are not significant, and progression-free survival generally does not exceed 8-10 months. PSA evaluation can be conducted monthly, as done by Barata et al., (2018). In addition, an early decrease of PSA was detected within the first four weeks in Yun et al., (2011)’ study. All of the analyzed studies reported a significant decrease in serum PSA ( >50%) in mCRPC patients receiving ketoconazole. Eight studies reported PSA response in >50% of patients, while Scholz et al., reported an even greater decrease of PSA (>75%) in 44% of patients. Chiang et al., (2012) reported a greater decrease in PSA in the group receiving mg ketoconazole at dose of 400, compared to the group receiving ketoconazole at dose of 200 mg.

Signs of toxicity must be closely monitored, and the treatment with ketoconazole should be ceased immediately if grade 3 or 4 toxicity occurred. In grades < 3 toxicity, the patients should be monitored for the signs, and this management should be continued until disease progression. 

In conclusion, ketoconazole with corticosteroid replacement can be used as an anti-androgenic treatment option for docetaxel-naïve mCRPC, with satisfactory PSA response and progression-free survival. 

## Author Contribution Statement

All authors discussed the results and contributed to the preparation of the final manuscript.
